# Natural posture versus standardized position for the measurement of cervical sagittal alignment on X-rays: a prospective cohort study

**DOI:** 10.1016/j.bas.2026.107725

**Published:** 2026-11-02

**Authors:** Esther van Santbrink, Valérie Schuermans, Anouk Smeets, Rob de Bie, Henk van Santbrink, Toon Boselie

**Affiliations:** a Dept. of Neurosurgery, Maastricht University Medical Centre, Maastricht, the Netherlands; b Dept. of Neurosurgery, Zuyderland Medical Centre, Heerlen, the Netherlands; c CAPHRI Institute for Public Health and Primary Care, Maastricht University, Maastricht, the Netherlands; d Department of Epidemiology, Maastricht University, Maastricht, the Netherlands

**Keywords:** Cervical spine, Cervical sagittal alignment, C2-C7 Cobb angle, Body posture, Standardized position, X-ray

## Abstract

**Introduction:**

Cervical sagittal alignment has been a key parameter for evaluating risk factors and outcomes in cervical spine pathology and surgery. This is generally measured on lateral X-rays. Reported normative alignment values vary widely between and within individuals. Body positioning throughout imaging procedures may contribute to this variability.

**Research question:**

This study investigated the influence of body position on cervical sagittal alignment measured on lateral X-rays in asymptomatic individuals.

**Methods:**

In this prospective cohort study, 20 asymptomatic individuals were included and received cervical and whole spine lateral X-rays in two standing positions at two time points, 6 to 8 weeks apart. The first position was a natural uninstructed posture (P1), and the second position was standardized, with head, arms, legs, and feet in an instructed position (P2). The primary outcome was the difference in C2-C7 Cobb angle.

**Results:**

Mean difference in C2-C7 Cobb angle between both positions was 6.9° ± 5.5° (p < 0.001). Gore angle, Harrison posterior tangent angle, C2–C7 sagittal vertical axis, McGregor angle, C0–C2 angle, and C0–C7 angle were also significantly different between the two positions. Mean intraobserver ICCs were excellent and mean interobserver ICCs were good.

**Discussion and conclusion:**

Positioning during imaging of the cervical spine significantly influences cervical sagittal alignment and is of such a magnitude that it can induce a shift from lordosis to kyphosis. Consequently, cervical sagittal alignment values reported to date may be confounded. Defining patient positioning is essential to ensure reliable and comparable outcome measurements in both research and clinical settings.

## Highlights

• Positioning significantly influences cervical sagittal alignment measured on lateral X-rays.
• This influence can be of such magnitude that it can induce a shift from lordosis to kyphosis.
• Defining of an imaging protocol is essential to improve longitudinal comparability and support accurate interpretation of cervical sagittal alignment.
• We recommend the natural standing posture for the assessment of cervical and global sagittal balance parameters.

## 1 Introduction

Sagittal alignment parameters have generated increasing attention in cervical spine research over the past decades (Lee et al., 2020). Cervical alignment in the sagittal plane is one of the most used outcome parameters assessing cervical spine balance and is widely relied upon in both clinical decision-making and research. Interestingly, studies investigating normative values of cervical alignment in asymptomatic populations have reported large variations, with mean values ranging from .6 to 24.2° lordosis (Lee et al., 2020).

Differences in measurement techniques have been suggested to influence these reported values. Several methods have been described for assessing cervical alignment, including the C2–C7 Cobb angle, the Gore angle (GA), and the Harrison posterior tangent angle (HPTA) (Scheer et al., 2013). High reliability has been reported for all three methods (Janusz et al., 2016). Although the HPTA is considered more representative of overall cervical alignment, the C2–C7 Cobb angle and the GA are more user-friendly and therefore more commonly applied (Harrison et al., 2000).

The wide range of reported normative values also appears to be influenced by patient characteristics such as age and sex, with increasing age and male sex correlated with a greater cervical lordosis (Rossanez et al., 2023; Tang et al., 2019; Kim et al., 2014; Tao et al., 2021; Liu et al., 2015). Other factors, including muscle strength, global spinal alignment, and body posture during X-ray acquisition, have also been suggested to influence cervical sagittal alignment parameters (Tamai et al., 2019; Fehr et al., 2023; Ferrero et al., 2021; Le Huec et al., 2015). In contrast to the other above-mentioned factors, body posture represents a variable that is amenable to standardization.

Multiple studies have investigated the effects of head, arm, and leg positions on cervical alignment in the sagittal plane. Among the positions investigated, the natural body posture is often defined as a standing or sitting position with the arms positioned alongside the body and the head in a relaxed head position. However, this position obstructs visualization of the thoracic and lumbar spine, which limits investigations of the global spinal alignment (Park et al., 2015; Marks et al., 2003). Standardized positioning offers an alternative approach for visualization of the whole spine. One commonly used method is the standing position in which the arms are placed in the fists-on-clavicles position while maintaining a horizontal gaze (Vedantam et al., 2000; Aota et al., 2009, 2011; Horton et al., 2005). Alteration of arm position in the fists-on-clavicles position has been shown to significantly affect cervical alignment compared with a natural posture (Hey et al., 2021; Lee et al., 2021). A recent systematic review confirmed that positioning significantly influences cervical alignment, with reported differences between positions ranging from 1 to 16.6° (van Santbrink et al., 2025). Despite this consistently observed influence, available studies are limited and highly heterogeneous, and the optimal position for analyzing cervical sagittal alignment has not been established.

This study investigates the influence of positioning on cervical sagittal alignment measured on lateral X-rays in asymptomatic individuals aged 18 to 75 years.

## 2 Methods

A prospective cohort study was performed at Zuyderland Medical Center (ZMC), the Netherlands, a tertiary spine center. This study was approved by the Medical Ethics Review Committee of ZMC (NL-05315/NL87591.096.24, METCZ20240087). The study was conducted according to principles enshrined in the Declaration of Helsinki and in accordance with the Medical Research Involving Human Subjects Act (WMO). Informed consent was acquired from all participants before start of the study.

Participants between 18 and 75 years of age, without current complaints of neck or back pain and without a history of spine surgery, trauma to the spine, congenital spinal abnormalities, metabolic bone diseases, inflammatory spinal pathology, or spinal oncology, were included. Study participants underwent cervical and whole spine lateral X-ray examinations in two different positions at two time points (T1 and T2), with an interval of 6 to 8 weeks. The study flowchart is shown in Fig. 1.

**Fig. 1:**
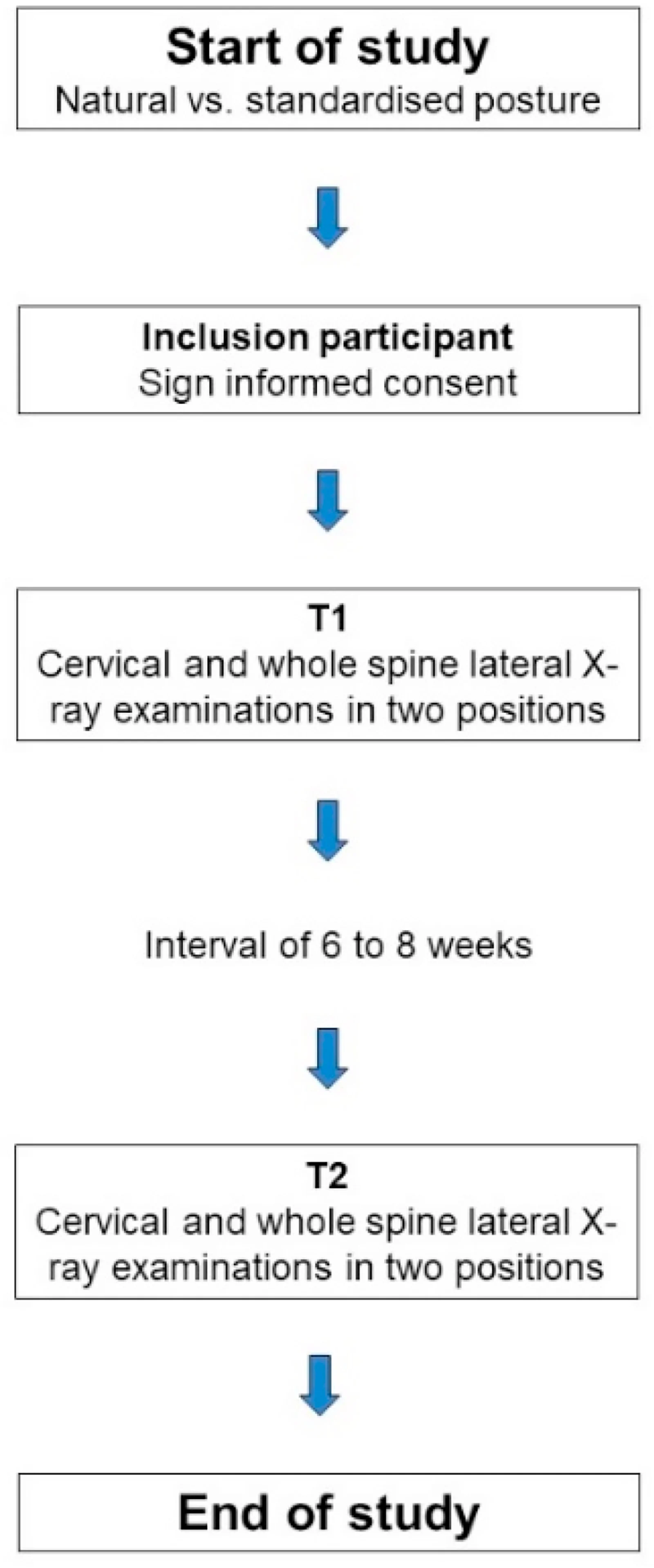
Flowchart of study process.

### 2.1 Investigated positions

In both positions, participants stood on a platform with external support for the hands. This was required capturing the whole spine lateral X-ray, in which separate images of the cervical, thoracic, and lumbar spine were fused into one image.

The first position was specified as a natural standing posture (P1, Fig. 2a). Participants stood barefoot in a relaxed manner, with their hands loosely positioned on the external support, and maintained a horizontal gaze.

**Fig. 2:**
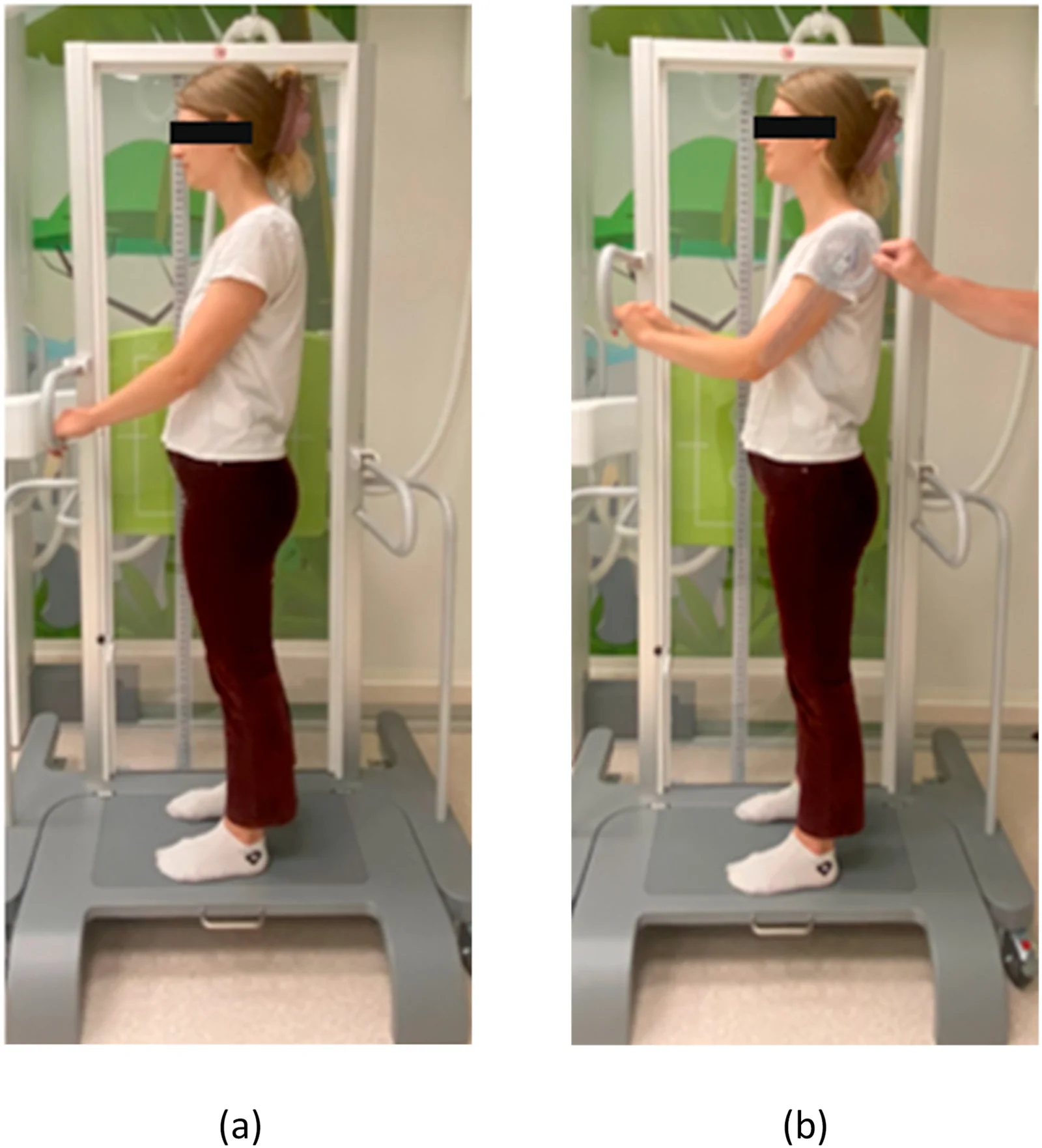
Example of the investigated positions: (a) P1: natural posture; (b) P2: standardized position: upper arms ensured with a goniometer in 30°, knees locked, and head fixated by mirror placement.

The second position was standardized (P2, Fig. 2b). Participants were guided using verbal and visual instructions to maintain the correct position. They were instructed to look into their own eyes, which was facilitated by placing a mirror in front of them. The mirror was placed at a distance of 1.0 m in front of the participant. The upper arms were held in forward anteflexion at 30° with flexed elbows and hands placed on the external support. External support was adjusted accordingly, and the upper arm position was checked with a goniometer. Feet were positioned at shoulder width with knees in a locked position. The beam-detector distance was 1.10 m for the cervical X-ray and 2.60 m for the whole spine X-ray. X-rays were made with a Philips Allura Xper FD20 X-ray system, capturing frames of 1024 × 1024 pixels.

The sequence of positions was consistent for all participants: the natural standing posture was performed first, followed by the standardized position. This approach was used to prevent memorization of the standardized position and therefore, eliminate its influence.

### 2.2 Sample size calculation

To achieve a power of 80% and a level of significance of 5% (two-sided), the study required a sample size of 40 participants to detect that the mean of the paired differences of the C2-C7 Cobb angle significantly differs from zero (Dhand and Khatkar). Assuming a 5% loss to follow-up after T1, the required sample size was 42 participants.

Per protocol, an interim analysis after 20 inclusions was performed to check for efficacy using the Snapinn stopping rule with a significance level of 5%, a statistical power of 80%, and an efficacy boundary probability of 90%.

### 2.3 Main study endpoints

The primary outcome was the absolute difference in cervical alignment in the sagittal plane, defined as the C2-C7 Cobb angle, measured in both positions. Secondary outcomes included the differences in the GA, HPTA, C2–C7 sagittal vertical axis (C2–C7 SVA), C0–C2 angle, C0–C7 angle, McGregor slope (McGS), T1 slope (T1S, and neck tilt (NT), measured in the two positions. The differences in thoracic kyphosis (TK), pelvic incidence (PI), pelvic tilt (PT), sacral slope (SS), and C7 sagittal vertical axis (SVA) for the two positions were investigated with whole spine lateral X-rays.

Intra-individual variability was assessed by acquiring X-rays in both positions at two time points, 6 to 8 weeks apart. Intra- and interobserver reliability were evaluated using repeated measurements, with two independent observers assessing all measurements, and one observer repeating all measurements at T1 after a 4-week interval. Sex, age, height and weight were gathered at baseline.

### 2.4 Radiographic measurements

The C2-C7 Cobb angle is measured from the inferior endplate of C2 to the inferior endplate of C7, and expressed in number of degrees (Figure A1, Appendix 1).

Secondary outcomes were the GA, HPTA, C2-C7 SVA, McGS, C0-C2 angle, T1S, NT, TK, C7 SVA, PI, PT, and SS (Figures A2-A5, Appendix 1). The GA is the angle between two lines drawn at the two posterior vertebral body corners of C2 and C7. The HPTA is the sum of the segmental angles between the lines that are drawn tangent to the two posterior vertebral body corners of vertebrae C2 to C7. The C2-C7 SVA is the horizontal distance from the C2 plumb line (origin mid-vertebrae C2) to the posterosuperior corner of C7 vertebrae, expressed in millimeters (mm). The McGS is the angle of the line from the posterior part of the hard palate to the most caudal point of the occipital bone (the McGregor line) in relation to a horizontal line. The C0-C2 angle is the angle between the McGregor line in relation to the line of the inferior endplate of C2. The C0-C7 angle is the angle between the McGregor line in relation to the line of the inferior endplate of C7. The T1S is the angle of the line of the superior endplate of the first thoracic vertebra (Th1) in relation to a horizontal line. The NT angle is measured from the midpoint of the Th1 superior endplate to the cranial point of the sternum in relation to a vertical line. The TK is the Th4-Th12 Cobb angle measured from the superior endplate of Th4 to the inferior endplate of Th12. The C7 SVA is the horizontal distance from the C7 plumb line (origin mid-vertebrae C7) to the posterosuperior corner of S1 vertebrae, expressed in millimeters (mm). The PT is the angle between a line from the axis of the femoral heads to mid sacral plateau in relation to a vertical line. The SS is the angle of the line of the sacral plateau in relation to a horizontal line. The PI is the sum of the PT and SS.

### 2.5 Statistical analysis

Participants’ characteristics and measurements are presented as means and standard deviation (±SD) for continuous variables and absolutes and percentages (%) for categorical variables.

For comparison of all measurements in the two positions, a paired *t*-test was used if the data was normally distributed. To determine if a normal distribution is present, a skewness and kurtosis test was performed. If normal distribution was not present, a Wilcoxon signed-rank test was used.

To account for repeated measurements and evaluate the influence of time, linear mixed-effects model (LMM) with participant as a random effect was additionally performed.

Within-subject variability between the two measurement sessions (T1 and T2) was assessed separately for each position by calculating the absolute T1–T2 difference for each sagittal alignment parameter. These absolute differences were compared between natural posture and standardized positioning using paired-samples t-tests or Wilcoxon signed-rank tests.

ICCs were calculated to determine interobserver and intraobserver reliability for all included outcome parameters. A two-way mixed effects model, testing for absolute agreement, was used. Values less than .4 were rated as poor reliability, values between .4 and .6 as moderate, values between .6 and .8 as good, and values larger than .8 as excellent (Landis and Koch, 1977).

A two-sided p-value of < .05 was considered statistically significant. SPSS 28.0 or higher software was used (IBM SPSS Statistics for Windows, 2021).

## 3 Results

Following the Snapinn stopping rule, when 50% of the originally planned study population was included, the study was stopped for efficacy if the p-value is < .0069 (H.J.A. S, 1999). This criterion was met, and the planned sample size was consequently amended, reducing the target inclusion from 40 to 20 participants in accordance with the results of this analysis.

Mean age was 38 years (SD ± 14), and 60% of participants were female. Mean time between T1 and T2 was 54 days (SD ± 14). Participant characteristics can be found in Table 1.

**Table 1:** Participants’ characteristics at baseline.

Characteristic	n = 20
Male (%)	8 (40)
Female (%)	12 (60)
Mean Age in years (SD)	38 ± 14
Average Height in cm (SD)	173 ± 9
Average Weight in kg (SD)	76 ± 13
Mean BMI in kg/m^2^ (SD)	25 ± 3
Mean time between imaging in days (SD)	54 ± 14

The mean difference in C2-C7 Cobb angle between the two positions was 6.9° ± 5.5° (p < 0.001). Significant differences between positions were observed for Cobb C2–C7 angle, GA, HPTA, C2–C7 SVA, McGS, C0–C2 angle, and C0–C7 angle (p < 0.05). T1S, NT, C7 SVA, SS, PT, PI, and TK did not significantly differ between the two different positions. Table 2 summarizes absolute differences in sagittal alignment parameters measured in the natural posture and standardized position from both T1 and T2. Fig. 3, Fig. 4 illustrate individual cases of a shift in cervical alignment due to positioning. Fig. 5 presents boxplots of the Cobb C2–C7 angle in the natural posture and standardized position.

**Table 2:** Absolute differences in parameters between natural posture and standardized position.

Parameter	Mean difference (SD)	95% CI	p	n
***Cobb C2-C7 (°)***	6.9 (5.5)	4.4 – 9.5	**<0.001**	20
***Gore Angle (°)***	6.6 (5.0)	4.3 – 9.0	**<0.001**	20
***Harrison posterior tangent angle (°)***	6.2 (4.6)	4.1 – 8.4	**<0.001**	20
***C2-C7 SVA (mm)***	−3.5 (4.8)	−5.7 to −1.2	**0.004**	20
***McGregor angle (°)***	2.3 (4.5)	.2 – 4.4	**0.035**	20
***C0-C2 angle (°)***	−1.2 (2.4)	−2.3 to −.1	**0.038**	20
***C0-C7 angle (°)***	6.0 (5.1)	3.5 – 8.4	**<0.001**	20
T1 slope (°)*	−.2 (4.2)	−2.7 – 2.4	.897	13
Neck Tilt (°)*	−.8 (2.8)	−3.1 – 1.5	.431	8

***C7 SVA (mm)***	.1 (15.3)	−7.1 – 7.2	.983	20
***Sacral Slope (°)***	−1.1 (3.3)	−2.6 – .4	.151	20
***Pelvic Tilt (°)***	.8 (2.6)	−.4 – 2.0	.190	20
***Pelvic Incidence (°)***	1.2 (4.7)	−1.1 – 3.4	.294	19
Thoracic kyphosis (°)*	−.7 (12.6)	−8.3 – 7.0	.855	13

The absolute difference was calculated using measurements obtained at T1 and T2.

**Fig. 3:**
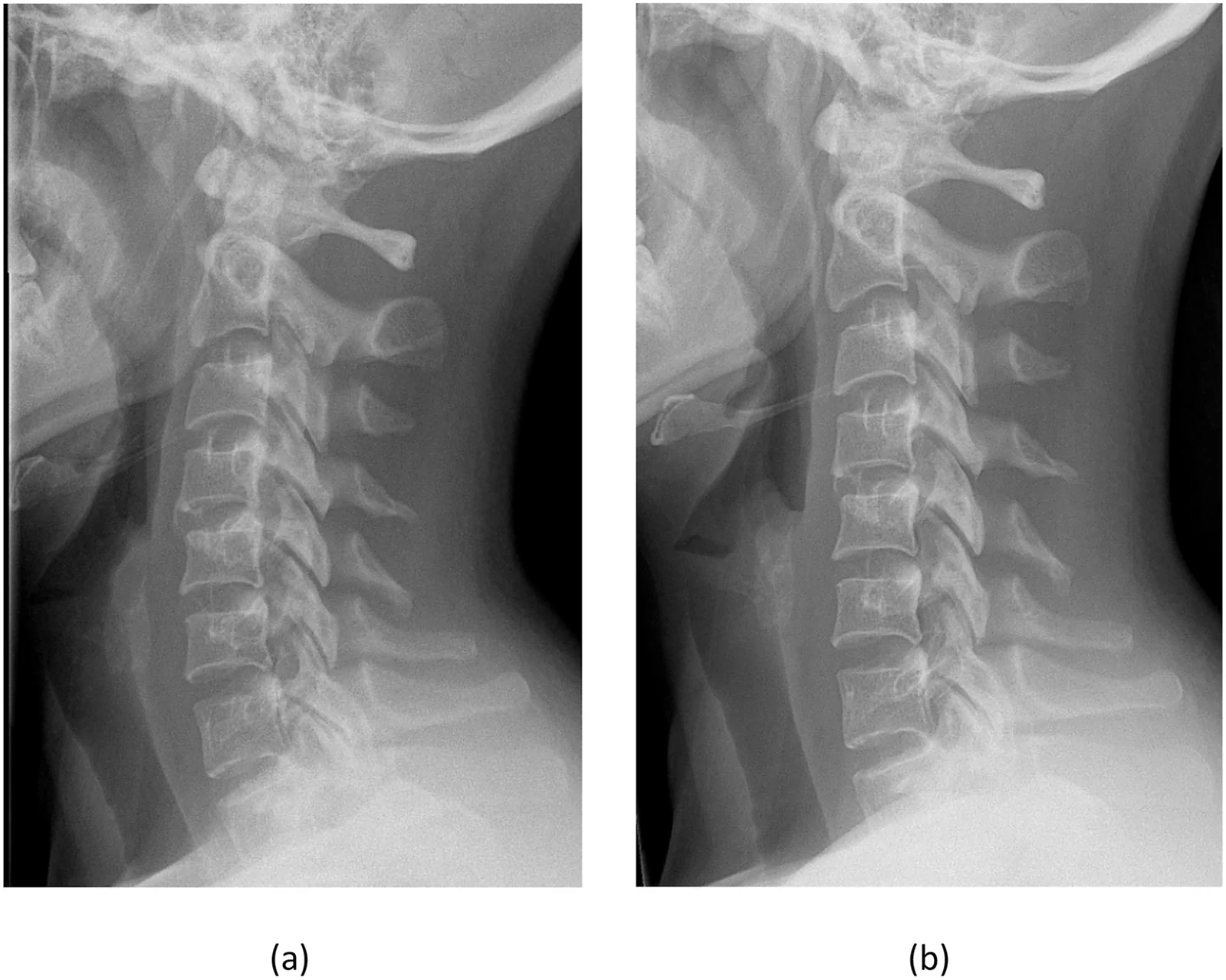
Example of altering cervical alignment from 6° lordosis to 10° kyphosis due to positioning: (a) natural posture, (b) standardized position.

**Fig. 4:**
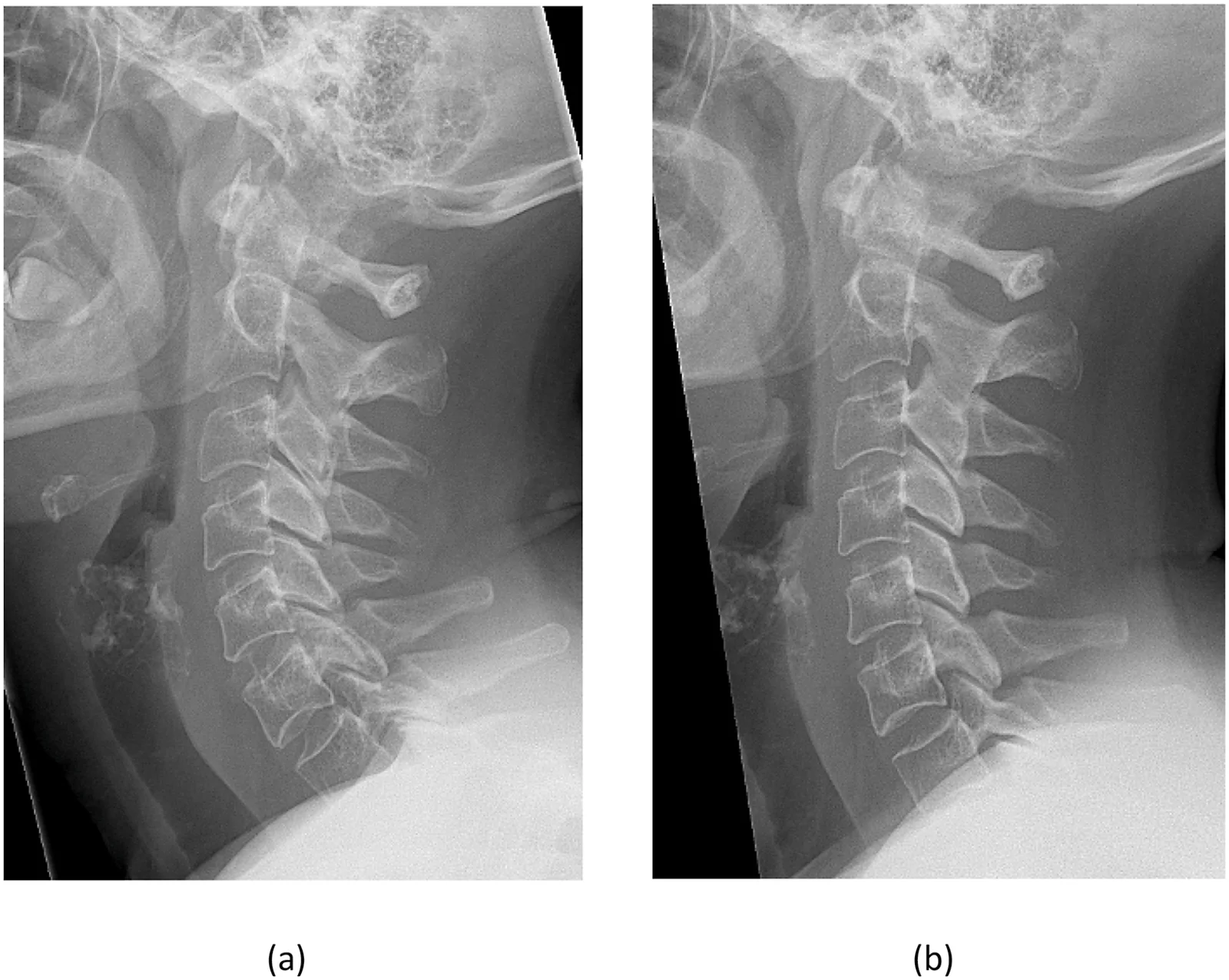
Example of altering cervical alignment from 39° lordosis to 19° lordosis due to positioning: (a) natural posture, (b) standardized position.

**Fig. 5:**
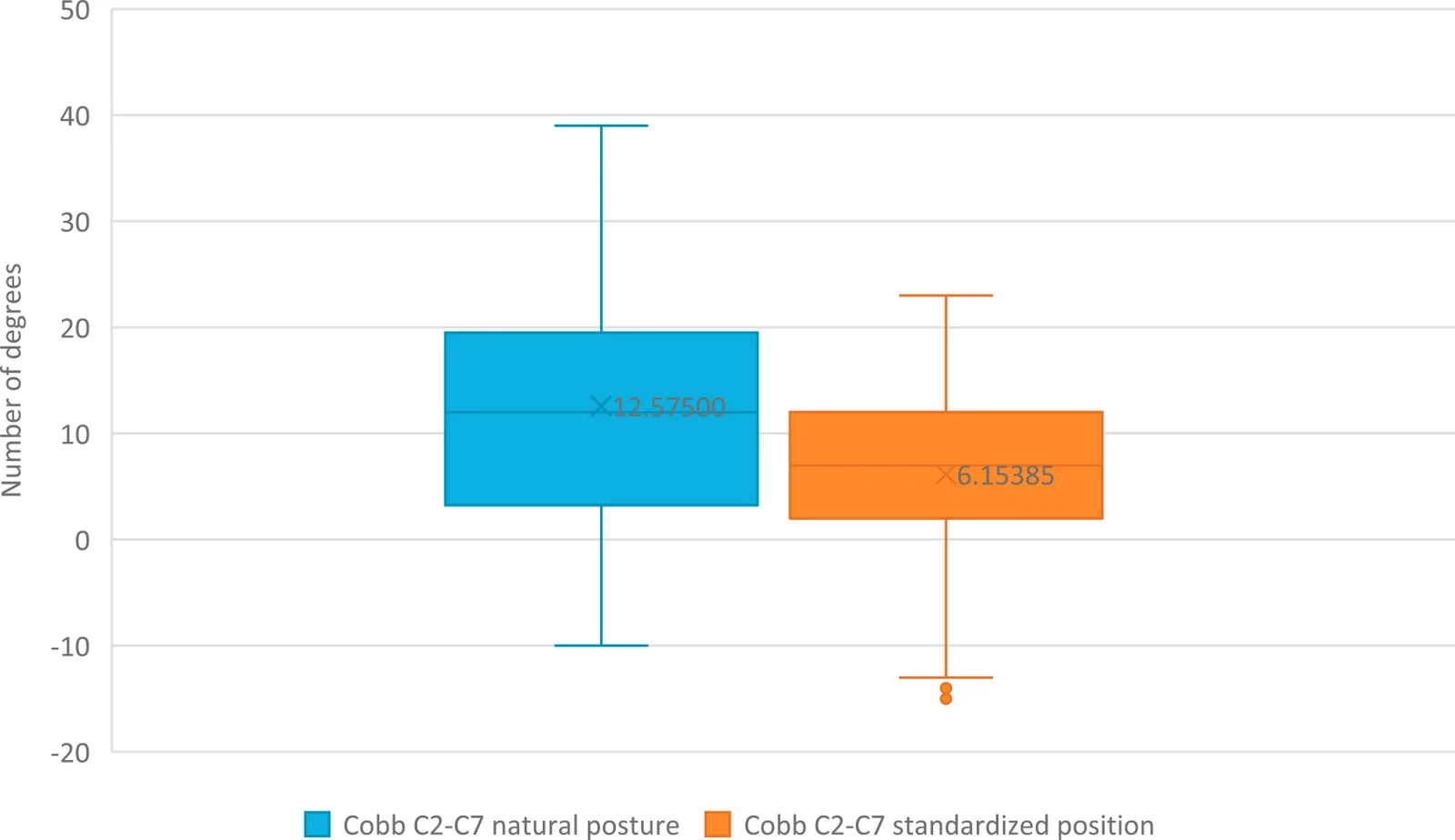
Boxplot Cobb C2–C7 angle measured in natural posture and standardized position, based on raw data.

At T1 mean intraobserver ICCs were excellent (P1 = .961; P2 = .940), while mean interobserver ICCs indicated good agreement (P1 = .871; P2 = .820).

An overview of all measurements of T1 and T2 can be found in Table A1 and Table A2, Appendix 1.

Linear mixed-effects modeling confirmed a significant effect of positioning (p < 0.001). Measurements did not differ significantly between time points (p = 0.084), and no position-by-time interaction was observed (p = 0.49).

Within-subject variability between T1 and T2 was assessed for both natural posture and standardized positioning (Table A3). Significantly greater variability was observed for the C2–C7 Cobb angle, GA, and McGS in natural posture compared with standardized positioning.

## 4 Discussion

Accurate assessment of cervical sagittal alignment is essential for both clinical decision-making and research. This study demonstrates that positioning exerts a significant influence on cervical sagittal alignment as measured on lateral X-rays. In individual cases, cervical alignment even shifted from lordosis in a natural posture to kyphosis in a standardized position (Fig. 3). This finding is particularly important, as much of the existing literature relies on cervical alignment parameters obtained without defined imaging protocols, raising concerns about the reliability of such data. The absence of an interaction between position and time indicates that the effect of positioning is consistent across time points and was not influenced by repeated measurements or participant familiarization. Defining positioning will lead to improved comparability across longitudinal assessments and reduced variability in measured values.

Park et al. investigated comparable patient positions and similarly reported greater cervical lordosis in the natural posture (Park et al., 2015). The differences in Cobb angle (5.62°) and Gore angle (7.31°) were comparable to those observed in our study (6.9° and 6.6°, respectively). Consistent with these findings, Hey et al. also reported significantly greater cervical lordosis in the natural compared with the standardized position (7.42° vs. −3.51°, compared with 14.5° vs. 6.5° in our study; both p < 0.001) (Hey et al., 2021). Notably, their mean C2–C7 Cobb angle shifted from kyphosis in the standardized position to lordosis in the natural position.

These posture-induced differences are clinically relevant, as cervical sagittal alignment parameters play an important role in surgical decision-making, such as the selection between anterior and posterior approaches or between laminectomy with or without instrumentation (Cabraja et al., 2010; Teo et al., 2020). Given that these surgical strategies differ substantially in terms of invasiveness, biomechanical consequences, and postoperative recovery, accurate and reproducible measurements are essential. Inappropriate surgical selection may carry important clinical implications, including prolonged hospitalization or recovery, increased healthcare costs, and an increased risk of perioperative complications (Du et al., 2013; Warren et al., 2013).

The observed and significant differences in cervical sagittal alignment were independent of measurement technique, including the Cobb C2-C7 angle, HPTA, and GA. However, the HPTA and GA yielded relatively higher lordotic angles compared with the Cobb C2–C7 angle. While the HPTA is considered to most accurately reflect sagittal alignment, its assessment is technically demanding (Harrison et al., 2000). The GA provides a practical alternative with comparable measurement outcomes. In addition, the GA may be preferable to the Cobb C2–C7 angle, since visualization of the inferior endplate of C7 is frequently limited by overprojection of the shoulders, whereas the posterior vertebral body of C7 is more consistently identifiable. Similar limitations due to shoulder and arm overprojection were observed for other parameters. Overprojection onto Th1, the sternum and Th4 resulted in a limited number of measurable values for T1S, NT, and TK. These limitations were inherent to the investigated positions. For example, adequate visualization of the thoracic spine is typically achieved with the upper arms elevated to approximately 90°, rather than the upper arms elevated to 30°.

Interestingly, global spinal alignment parameters did not differ between the two positions. This suggests that posture-induced alterations in cervical alignment do not necessarily translate into changes in global spinal alignment. This may be explained by the fact that the standardized positioning protocol primarily controlled head and upper arm position. Moreover, the cervical spine exhibits substantially greater mobility than the thoracic and lumbar spine, while the pelvis and sacrum are largely rigid structures on which global spinal parameters are based. As a result, changes in cervical sagittal alignment may be accommodated within the cervical spine without substantially affecting global spinal alignment.

However, T1S, NT, PI, and TK were assessed in fewer than 20 subjects, which may have limited the statistical power to detect differences between positions.

Future studies should focus on defining imaging protocols. Both natural posture and the standardized position demonstrate high reliability for the assessment of sagittal alignment parameters. Although within-subject variability was slightly greater in natural posture for certain cervical parameters, both positions showed generally consistent measurements over time. In the natural posture, patients are instructed to maintain a horizontal gaze, and the arm position is minimally adjusted to improve visibility of the whole spine. This posture more closely reflects the patient's habitual daily posture. Therefore, we recommend assessing cervical and global sagittal alignment parameters in the natural standing posture. At a minimum, patient positioning during image acquisition should be clearly defined to allow for appropriate interpretation and comparison of results, especially between studies. Larger cohorts are needed to evaluate the influence of compensatory mechanisms.

## 5 Conclusion

Positioning significantly influences cervical sagittal alignment measured on lateral X-rays, and is of such a magnitude that it can induce a shift from lordosis to kyphosis. Consequently, cervical sagittal alignment values reported to date may be confounded by positioning. This study underscores the importance of defining imaging protocols to improve longitudinal comparability and support accurate interpretation of cervical alignment in both research and clinical decision-making.

## Author contributions

Conceptualization, E.v.S., V.S., A.S., R.d.B., H.v.S., and T.B.; methodology, E.v.S., V.S. and T.B..; software, E.v.S..; validation, E.v.S. and V.S.; formal analysis, E.v.S.; investigation, E.v.S..; resources, E.v.S. and T.B.; data curation, E.v.S..; writing—original draft preparation, E.v.S.; writing—review and editing, V.S., A.S., R.d.B., H.v.S., and T.B.; visualization, E.v.S..; supervision, V.S., A.S., R.d.B., H.v.S., and T.B.; project administration, E.v.S.

## Declaration of generative AI and AI-assisted technologies in the manuscript preparation process

During the preparation of this work the author(s) used ChatGPT in order to improve readability and language. After using this tool, the author(s) reviewed and edited the content as needed and take(s) full responsibility for the content of the published article.

## Funding

This research did not receive any specific grant from funding agencies in the public, commercial, or not-for-profit sectors.

## Declaration of competing interest

The authors declare the following financial interests/personal relationships which may be considered as potential competing interests: Henk van Santbrink reports a relationship with B Braun Medical Inc that includes: funding grants. If there are other authors, they declare that they have no known competing financial interests or personal relationships that could have appeared to influence the work reported in this paper.

## Figures and Tables

**Fig. A1:**
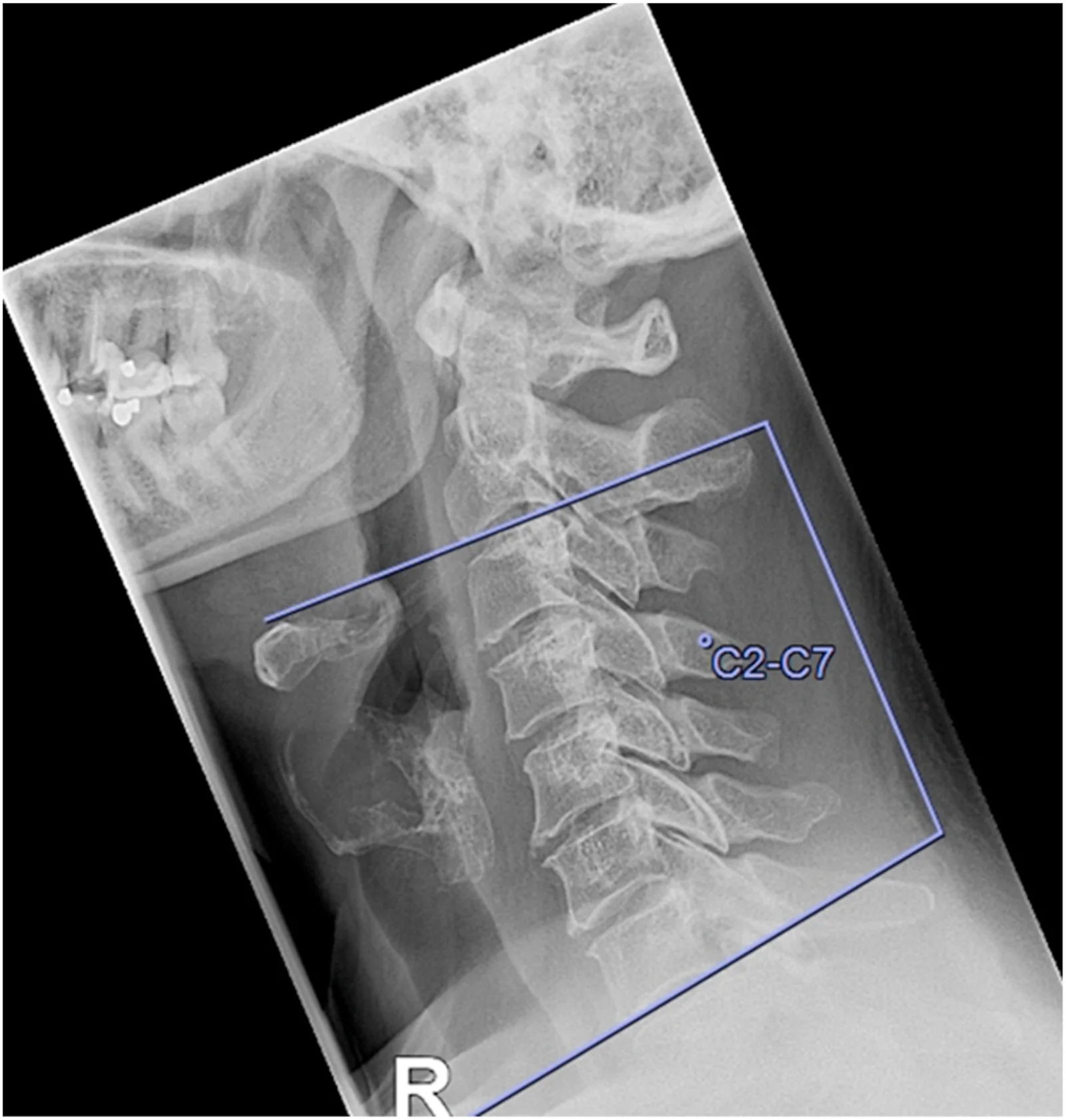
Cobb Angle measured from C2 to C7.

**Fig. A2:**
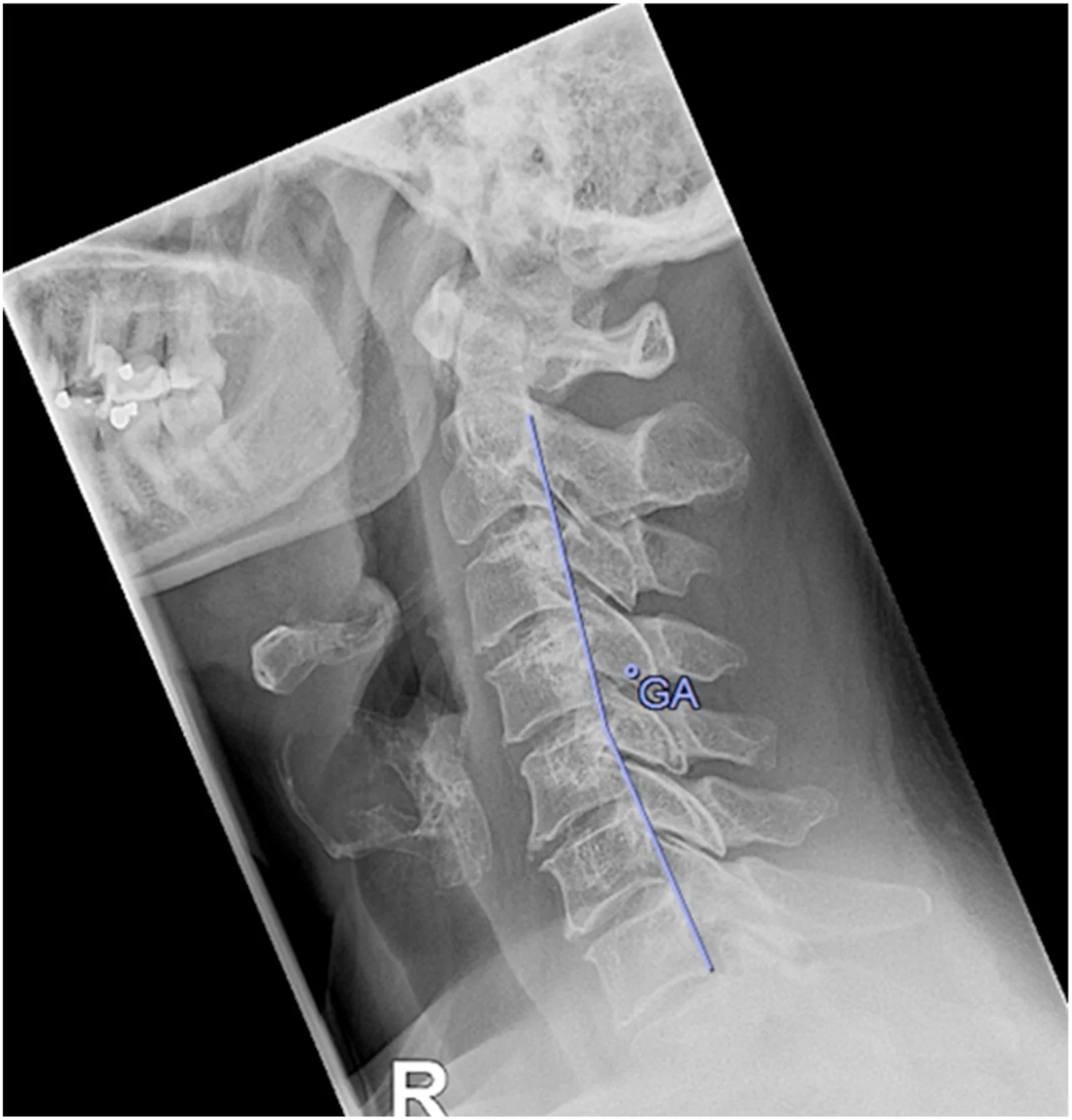
Gore angle, two lines drawn posterior of vertebral bodies C2 and C7.

**Fig. A3:**
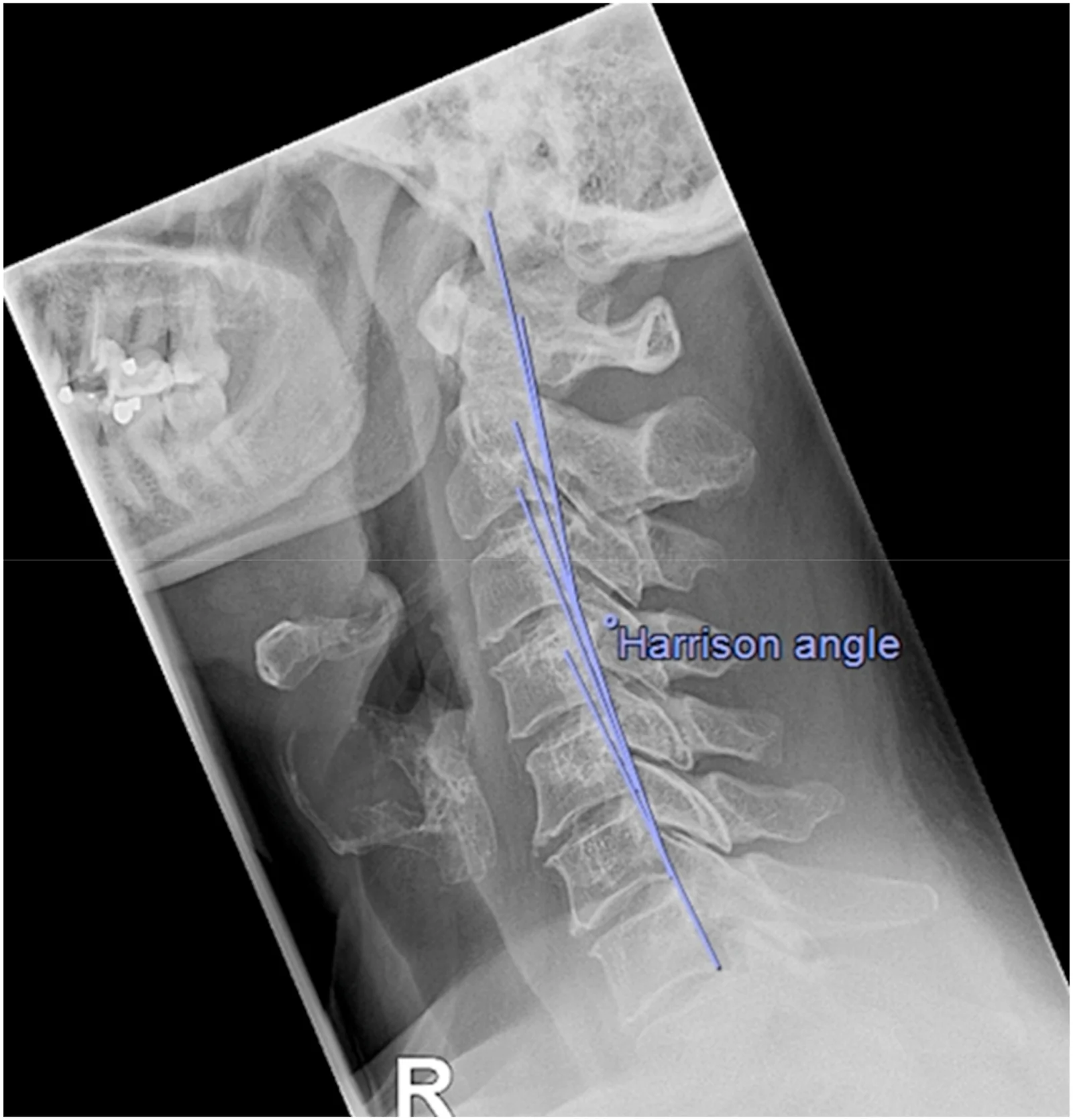
Harrison posterior tangent angle, lines drawn posterior of vertebral bodies C2 to C7.

**Fig. A4:**
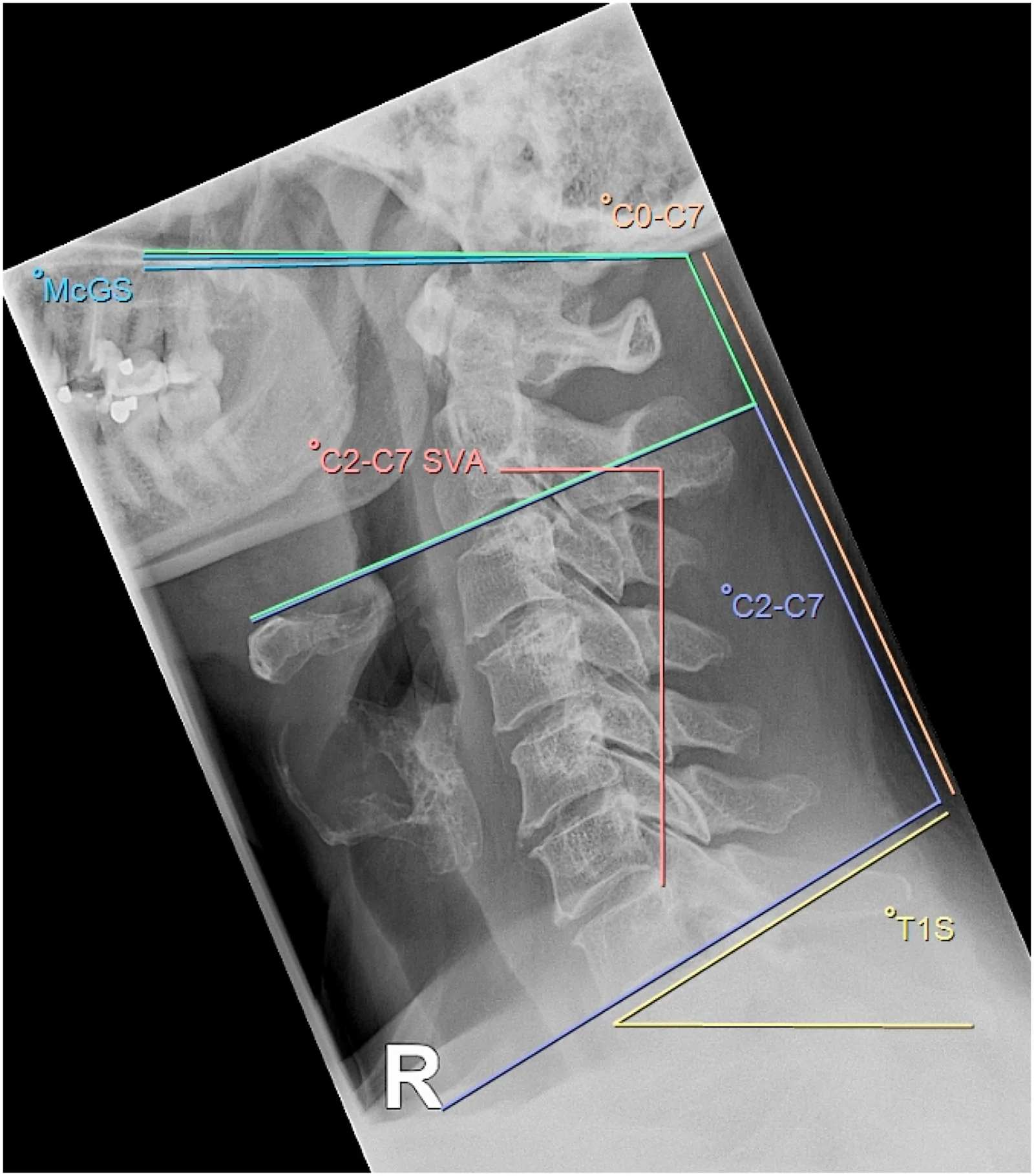
Cervical sagittal alignment parameters. Blue = McGregor Slope (McGS), orange = C0-C7 angle, red = C2-C7 sagittal vertical axis (C2-7 SVA), purple = C2-C7 angle, yellow = thoracic 1 slope (T1S).

**Fig. A5:**
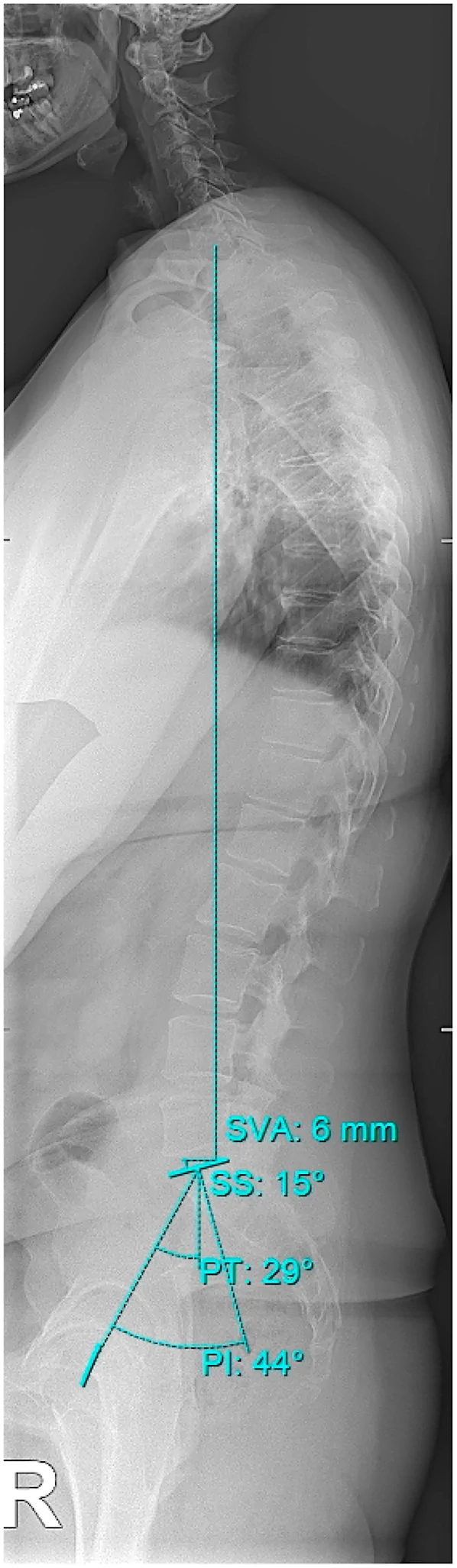
Global spinal alignment parameters. SVA = sagittal vertical axis, SS = sacral slope, PT = pelvic tilt, PI = pelvic incidence.

**Table A1:** Mean measurement outcome per position (T1) with inter-class correlation for absolute agreement, intra- and interobserver reliability.

	Natural posture (SD)	Standardized position (SD)	p	Intraobserver P1 (95% CI)	Intraobserver P2 (95% CI)	n	Interobserver P1 (95% CI)	Interobserver P2 (95% CI)	n
***Cobb C2-C7 (°)***	14.5 (11.6)	6.5 (10.4)	**<0.001**	.968 (.920-.987)	.987 (.968-.995)	20	.909 (.784-.963)	.851 (.666-.938)	20
***Gore Angle (°)***	22.2 (10.8)	13.7 (8.9)	**<0.001**	.967 (.920-.987)	.963 (.910-.985)	20	.939 (.854-.976)	.899 (.766-.959)	20
***Harrison posterior tangent angle (°)***	21.3 (10.8)	12.7 (9.0)	**<0.001**	.975 (.938-.990)	.967 (.890-.988)	20	.813 (.587-.922)	.698 (.382-.868)	20
***C2-C7 SVA (mm)***	31.4 (11.9)	36.1 (13.7)	**0.005**	.998 (.996-.999)	.997 (.957-.999)	20	.934 (.841-.973)	.980 (.653-.995)	20
***McGregor angle (°)***	.6 (4.9)	−2.4 (6.0)	**0.027**	.978 (.944-.992)	.984 (.886-.996)	18/19	.842 (.628-.938)	.960 (.901-.984)	18/19
***C0-C2 angle (°)***	15.4 (6.1)	16.9 (6.8)	.070	.933 (.830-.974)	.947 (.866-.979)	18/19	.618 (.226-.838)	.843 (.635-.936)	18/19
***C0-C7 angle (°)***	31.1 (12.0)	23.1 (11.4)	**<0.001**	.978 (.941-.992)	.979 (.943-.992)	18/19	.863 (.672-.947)	.900 (.763-.960)	18/19
***T1 slope (°)***	32.9 (6.0)	31.7 (4.2)	.474	.931 (.791-.978)	.667 (.181-.891)	13/12	.798 (.462-.934)	.668 (.213-.890)	13/12
***Neck Tilt (°)***	38.7 (10.8)	41.0 (6.9)	.285	.998 (.971-1.0)	.929 (.007-.998)	4/3	.994 (.778-1.0)	.179 (−.229-.757)	3/6

***C7 SVA (mm)***	17.3 (26.4)	19.2 (24.7)	.682	.999 (.997-1.0)	.996 (.990-.998)	20	.990 (.975-.996)	.986 (.965-.994)	20
***Sacral Slope (°)***	34.5 (7.5)	35.5 (7.1)	.285	.878 (.718-.950)	.895 (.755-.957)	20	.831 (.622-.930)	.791 (.551-.911)	20
***Pelvic Tilt (°)***	14.7 (5.6)	13.8 (6.3)	.192	.991 (.976-.996)	.957 (.896-.983)	20	.959 (.899-.983)	.961 (.907-.984)	20
***Pelvic Incidence (°)***	49.2 (8.5)	49.3 (8.7)	.889	.878 (.718-.950)	.926 (.824-.970)	20	.835 (.631-.931)	.848 (.657-.937)	20
***Thoracic kyphosis (°)***	44.6 (15.2)	41.6 (10.8)	.249	.983 (.937-.995)	.962 (.796-.992)	11/8	.869 (.587-.963)	.912 (.678-.979)	11/9

Wilcoxon signed-rank test was used because data was not normally distributed. P1 = natural posture, P2 = standardized position.

**Table A2:** Mean measurement outcome per position (T2) with inter-class correlation for absolute agreement, interobserver reliability.

	Natural posture (SD)	Standardized position (SD)	p	n	Interobserver P1 (95% CI)	Interobserver P2 (95% CI)	n
***Cobb C2-C7 (°)***	11.2 (10.0)	5.8 (7.3)	**<0.001**	19	.909 (.776-.964)	.916 (.717-.971)	20/19
***Gore Angle (°)***	17.8 (9.7)	12.7 (8.2)	**<0.001**	18	.571 (.172-.806)	.814 (.573-.926)	20/18
***Harrison posterior tangent angle (°)***	17.6 (9.9)	13.1 (8.4)	**0.002**	18	.671 (.346-.854)	.761 (.475-.903)	20/18
***C2-C7 SVA (mm)***	33.6 (11.8)	35.2 (12.1)	.204	18	.962 (.906-.985)	.944 (.740-.983)	20/18
***McGregor angle (°)***	−.6 (5.1)	−2.3 (5.4)	.182	20	.677 (.350-.858)	.916 (.797-.966)	20
***C0-C2 angle (°)***	16.5 (5.2)	17.6 (5.5)	.069	20	.869 (.698-.946)	.829 (.607-.929)	20
***C0-C7 angle (°)***	27.2 (10.4)	23.2 (8.6)	**0.008**	19	.942 (.860-.977)	.881 (.721-.952)	20/19
***T1 slope (°)***	32.1 (5.5)	33.1 (3.5)	.448	11	.432 (−.138-.785)	.298 (−.186-.721)	13/11
***Neck Tilt (°)***	36.8 (3.7)	36.8 (3.6)	1.0	5	.770 (-.073-.960)	.849 (.087-.979)	5/6

***C7 SVA (mm)***	17.8 (29.5)	15.3 (27.1)	.470	19	.985 (.961-.994)	.987 (.925-.996)	19
***Sacral Slope (°)***	34.4 (7.1)	35.6 (6.5)	.127	19	.758 (.347-.910)	.917 (.801-.967)	19
***Pelvic Tilt (°)***	14.5 (6.5)	13.8 (6.2)	.354	19	.930 (.828-.972)	.964 (.679-.990)	19
***Pelvic Incidence (°)***	48.8 (7.9)	49.6 (7.8)	.197	19	.834 (.343-.947)	.900 (.689-.964)	19
***Thoracic kyphosis (°)***	45.0 (11.0)	40.8 (10.4)	**0.011**	8	.863 (.566-.963)	.952 (.809-.989)	10/9

P1 = natural posture, P2 = standardized position.

**Table A3:** Within-subject variability in sagittal alignment parameters between T1 and T2 for natural posture and standardized position.

	Natural posture (SD)	Standardized position (SD)	Mean paired difference (95% CI)	p	n
***Cobb C2-C7 (°)***	7.1 (5.2)	3.8 (3.1)	3.6 (1.0-6.2)	**0.009**	19
***Gore Angle (°)***	7.1 (5.3)	3.3 (3.4)	3.4 (.2-6.6)	**0.040**	18
***Harrison posterior tangent angle (°)***	6.6 (5.4)	3.8 (3.1)	2.3 (−.8-5.4)	.129	18
***C2-C7 SVA (mm)***	5.8 (3.4)	3.9 (3.3)	1.8 (−.8-4.4)	.170	18
***McGregor angle (°)***	4.7 (2.8)	2.3 (2.5)	2.2 (.2-4.1)	**0.031**	18
***C0-C2 angle (°)***	2.4 (1.9)	3.2 (2.0)	−.5 (−1.6-.6)	.326	18
***C0-C7 angle (°)***	5.5 (4.2)	3.5 (3.1)	2.5 (−.2-5.2)	.064	18
***T1 slope (°)***	4.8 (2.2)	2.5 (2.7)	2.0 (−.7-4.7)	.128	10
***Neck Tilt (°)***	3.0 (3.0)	6.5 (6.4)	-	-	2

***C7 SVA (mm)***	13.7 (13.2)	15.7 (10.7)	−1.9 (−9.7-5.8)	.604	19
***Sacral Slope (°)***	3.4 (2.7)	3.7 (2.6)	−.3 (−1.7-1.1)	.648	19
***Pelvic Tilt (°)***	2.8 (2.2)	2.6 (1.5)	.2 (−.8-1.3)	.678	19
***Pelvic Incidence (°)***	1.6 (1.5)	2.6 (2.0)	−1.1 (−1.8 to −.4)	**0.014**	19
***Thoracic kyphosis (°)***	3.4 (2.2)	2.3 (2.0)	1.0 (−2.5-4.5)	.515	7

Positive values indicate greater within-subject variability in natural posture.

Wilcoxon signed-rank test was used because data was not normally distributed.
